# On a collision course: fatal motorcycle and bicycle accidents of adolescents in Finland from 2008 to 2019

**DOI:** 10.1093/eurpub/ckad198

**Published:** 2023-11-08

**Authors:** Jani Unkuri, Päivi Salminen, Niina Sihvola, Silja Kosola

**Affiliations:** Pediatric Surgery, New Children’s Hospital, Helsinki University Hospital and University of Helsinki, Finland; Pediatric Surgery, New Children’s Hospital, Helsinki University Hospital and University of Helsinki, Finland; Finnish Crash Data Institute, Helsinki, Finland; Pediatric Research Center, New Children’s Hospital, Helsinki University Hospital and University of Helsinki, Finland; Research, Development and Innovations, Western Uusimaa Welfare Services County, Finland

## Abstract

**Background:**

Transport injuries are a major cause of mortality among adolescents. Our aim was to evaluate the circumstances and trauma associated with fatal accidents involving adolescents and two-wheeled vehicles.

**Methods:**

We analyzed retrospective data from the Finnish Crash Data Institute from 2008 to 2019 involving 10- to 24-year-old victims of fatal traffic accidents who were injured while riding a bicycle, moped or motorcycle. We collected data on patient characteristics, accident circumstances and possible treatment. These fatalities were compared with national mortality rates among the respective age groups.

**Results:**

We identified 147 fatalities over the 12-year period involving 20 bicycle, 50 moped and 77 motorcycle riders. Most accidents involved males (*n* = 121, 82%). Less than half of vehicles were in good condition (46%); motorized vehicles were often illegally tuned (37%) or had tire problems (31%). Most of the accidents were collisions with another vehicle (*n* = 99, 67%) or other objects (*n* = 35, 24%). In 94% of cases, the Injury Severity Score was >25. Head injury was the most common cause of death (62%). Among 15-year-olds, every fifth death was due to accidents on two-wheeled vehicles.

**Conclusions:**

Fatal transport accidents among adolescents comprise several elements that should be incorporated into driver’s education and in case of minors, also communicated to parents. These include the condition of the vehicle, proper helmet use and effects of speed on both control of the vehicle and the consequences of a possible collision.

## Introduction

Adolescents desire autonomy in all areas of their lives, including transportation. In high-income countries, transport injuries and suicides are unfortunately among the leading causes of death among adolescents aged 10–24.^1–3^ The highest transport-related death rates among 15- to 19-year-old adolescents in the EU-27 countries are reported in Lithuania, Finland and Norway.^4^ While mortality rates among young children have improved markedly, much slower positive development has been seen in adolescent mortality.^2^^,^^5^

Although the rates of fatal transport accidents involving adolescents have decreased in Europe during the last two decades, circa 5700 young people aged 15–29 were killed in transport accidents in the EU-27 countries in 2016.^6^^,^^7^ The most common critical event leading to both fatal and non-fatal accidents is speeding.^7–9^ Other well-known risk factors include young age, male sex, and driving under the influence (DUI) of alcohol or other substances.^8^

Every year, 2160 cyclists are killed in Europe, with no reduction since 2010.^10^ In adolescents, estimating speed and distance reach adult maturity by circa 12 years of age depending on exposure, and response times reach adult levels by circa 14 years.^11^ Bicycling is still relatively safe. In eight European countries, cyclist deaths ranged between 10–24 per billion kilometers cycled.^10^ In our previous study, the incidence of bicycle-related injuries requiring specialist treatment ranged from 90/100 000–180/100 000 among 7- to 15-year-olds.^12^

Mopeds and motorcycles offer another form of independent transport for adolescents and the popularity of mopeds and motorcycles is persistent in the European Union. The number of registered mopeds has remained constant (13.1 million in 2007 and 12.6 million in 2017) and the number of registered motorcycles has risen from 20.7 million in 2007 to 26.2 million in 2017.^13^ The estimated incidence of injuries requiring hospital treatment per new moped license was 1.0% in Finland in 2013.^14^ Previously recognized evidence gaps regarding powered two-wheelers include more in-depth investigations to increase understanding of causes and circumstances of fatal injuries.^15^

The aims of this study were to evaluate the circumstances and trauma associated with fatal accidents involving adolescents and two-wheeled vehicles, and to evaluate the burden of these fatalities on adolescent mortality.

## Methods

We utilized national data of fatal accidents involving 10- to 24-year-old bicycle, moped and motorcycle riders from years 2008 to 2019 from the Finnish Crash Data Institute (OTI) database. OTI is responsible for the maintenance of road accident investigation, the use of the investigation results and the information service.

According to Finnish law, driver’s license class AM includes mopeds with a maximum speed of 25–45 km h^−1^; class A1 motorcycles with a maximum cylinder capacity of 125 cubic cm (cc), maximum power of 11 kW and maximum power-to-weight ratio (PWR) 0.1 kW kg^−1^; class A2 motorcycles with a maximum power of 35 kW and maximum PWR 0.2 kW kg^−1^; and class A all other motorized two-wheelers.^16^ The respective minimum ages for acquiring a driver’s license are 15 for AM, 16 for A1, 18 for A2 and 24 for A (except if a person has had an A2 license for two years, 20 years). Due to the age limits, we grouped class A2 and A motorcycles together.

In Finland, all fatal motor vehicle accidents are studied in-depth on-the-spot by multidisciplinary road accident investigation teams.^17^ This investigation in regulated by legislation.^18^ OTI produces information and safety recommendations which are used to improve road safety. Currently, twenty road accident investigation teams exist nationally and include circa 280 members. Basic members of the teams are a traffic police officer, vehicle specialist, road safety specialist, physician and a behavioral scientist (usually a psychologist). Other experts may also be included when necessary (e.g. a railway specialist). The teams are independent and impartial during investigations and take no stand on guilt or insurance compensation. The investigation teams use an in-depth, on-the-spot case-study method.^19^

We gathered data on fatal bicycle, moped and motorcycle accidents of 10- to 24-year-olds between 2008 and 2019 from the OTI database and double-checked some details of accidents and injuries from the paper case folders of the road accident investigation teams.

Demographic data included age and sex. Driver and vehicle related data included vehicle type and condition, use of protective gear, and potential DUI. For accident circumstances, we collected data on the time and place of accidents, type of accident (and in case of collisions, type of counterpart), daylight and weather conditions, speed limit at the site of the accident, and estimated speeding. We also collected data on time from accident to death, place of death and potential operative treatment. The most serious injury was defined as the cause of death recorded in the autopsy report.

The Injury Severity Score (ISS) was used to assess the severity of the injury. ISS is one of the most common and widely used trauma scores and it correlates with mortality, morbidity and hospitalization time after the trauma.^20^ ISS divides the body into six different regions and takes into account the three most severely injured regions to calculate the score. Among adults injured in motor vehicle accidents, an ISS <16 has a negative predictive value of 99.8% for mortality, while higher ISS are associated with > 5% mortality rates.^21^ Among children, however, a similar mortality rate has been published when ISS was > 25.^22^ An ISS of 75 is considered untreatable trauma.

Since this study utilized register data from one database, no ethics approval was required.

Anonymous mortality rates from the OTI database were compared with national mortality statistics (available from Statistics Finland at stat.fi/index_en.html) to estimate the burden of deaths related to two-wheeled vehicles per age group.

Descriptive statistics are presented as frequencies, percentages and means (with standard deviation, SD). Fisher’s exact test was used for dichotomous variables. A *P* value < 0.05 was considered statistically significant. Statistical analyses were conducted using IBM SPSS Statistics version 25 (IBM, Somers, NY).

## Results

A total of 147 fatalities occurred among 10- to 24-year-old riders of bicycles, mopeds and motorcycles during the 12-year study period. Annual number of cases varied between 6 and 19 (Supplementary figure S1).

The mean age of the deceased was 17.0 years (SD 2.9) and most cases were males (*n* = 121, 82%) (table 1). Frequency of DUI increased with age. More than a quarter of riders used a helmet that was not attached properly. Less than half of vehicles were in good condition: all bicycles lacked proper lights and over half (56%) of the mopeds had been illegally tuned. Speeding was common especially among motorcycle riders. Accident sites were mostly in urban areas (47%) with a speed limit of 50 km h^−1^ or less; only 5% of the accidents occurred in highway settings with speed limits of 100 km h^−1^ or higher.

**Table 1:** Data on riders, vehicles and accident circumstances

	Bicycle (*n* = 20)	Moped (*n* = 50)	Motorcycle <125cc (*n* = 44)	Motorcycle >125cc (*n* = 33)	Total (*n* = 147)
Age, mean (SD)	15.5 (3.8)	15.5 (0.9)	17.0 (1.5)	20.2 (3.2)	17.0 (2.9)
Male sex, *n* (%)	11 (55)	40 (80)	40 (91)	30 (91)	121 (82)
DUI, *n* (%)	0	5 (10)	10 (23)	11 (33)	26 (18)
With passenger, *n* (%)	0	14 (28)	6 (14)	8 (24)	28 (19)
Helmet use, *n* (%)					
Yes, properly attached	2 (10)	29 (58)	31 (71)	21 (64)	83 (56)
Yes, but not properly attached	0	21 (42)	11 (25)	8 (24)	40 (27)
No	18 (90)	0	2 (5)	3 (9)	23 (16)
Vehicle condition, *n* (%)					
Good condition	19 (95)	14 (28)	19 (43)	14 (42)	66 (45)
Illegal tuning^a^	NA	28 (56)	17 (39)	10 (30)	
Tire problems^b^	0	18 (36)	14 (32)	14 (42)	46 (31)
Improper or missing lights	20 (100)	5 (10)	2 (5)	2 (6)	29 (20)
Other technical problems^c^	1 (5)	16 (32)	6 (14)	6 (18)	29 (20)
Estimated speed above legal limit, *n* (%)					
No	1 (5)	32 (64)	16 (36)	5 (15)	54 (37)
<10 km h^−1^	0	3 (6)	1 (2)	0	4 (3)
11–20 km h^−1^	0	6 (12)	5 (11)	4 (12)	15 (10)
>20 km h^−1^	0	5 (10)	20 (45)	21 (64)	46 (31)
Accident site, urban (%)	9 (45)	28 (56)	16 (36)	16 (49)	69 (47)

Notes: DUI, Driving under the influence (above legal limit of 5 g l^−1^ or evidence of drug use), SD, standard deviation. Data missing (*n*): DUI (12), with passenger (1), helmet use (1), vehicle condition (2), estimated speeding (9; speeding not estimated for bicyclists).

a Change in exhaust system and/or other modifications to increase the power of the engine and/or changing the sprockets to increase maximum speed.

b Tires worn below legal limits or inadequate tire pressure.

c For example, bad brakes, self-made tuning of the chassis.

Mondays, Fridays or Saturdays were the most common weekdays, but these differences were not statistically significant. Most accidents occurred in clear (*n* = 66, 45%) or cloudy (*n* = 67, 46%) weather, during daylight (*n* = 104, 71%), and in the afternoon or early evening.

**Figure 1:**
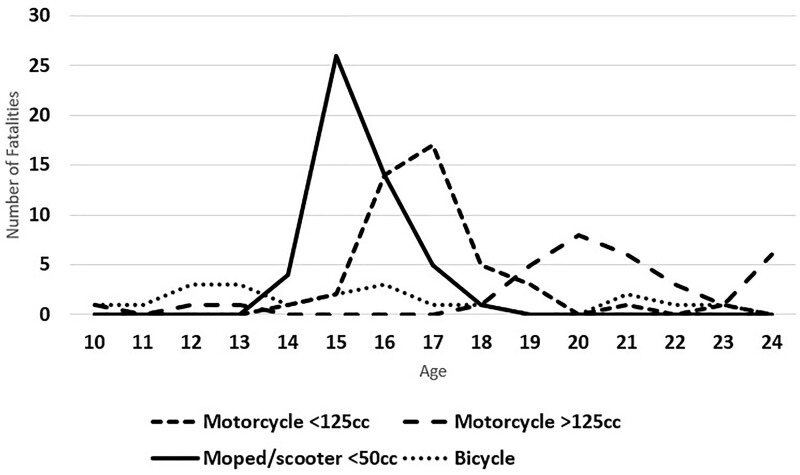
Fatalities of 147 adolescents according to age and vehicle type

The age distribution of rider deaths differed according to vehicle type (table 1, figure 1). Females less often rode a tuned vehicle or had technical flaws and less frequently engaged in speeding than did male riders, while males more often wore a helmet (table 2).

**Table 2:** Comparison of risk-taking behavior between females and males

	Females (*n* = 26)	Males (*n* = 121)	*P*
Illegal tuning	2 (8)	53 (44)	<0.001
Other problems besides tuning or tires	0	29 (24)	0.002
Speeding	3 (11)	62 (51)	0.001
Blood alcohol	1 (4)	19 (16)	0.196
Substance/medication use	2 (8)	24 (20)	0.145
No helmet	8 (31)	15 (12)	0.042

In two-thirds of accidents (*n* = 99), adolescents collided with another vehicle (see Supplementary table S1). Collisions accounted for a larger proportion of bicycle and moped rider fatalities than motorcyclist fatalities (*P* < 0.001).

The most common cause of death was a head injury (62%). In 94% of cases, the ISS was more than 25. Forty-one adolescents survived to the hospital and 19 of them (46%) underwent emergency operations, most commonly for a head injury. All deaths occurred within a week from the accident.

The proportion of two-wheeled vehicle fatalities among all-cause mortality differed greatly depending on age during the whole study period (table 3). The proportion was greatest among 15-year-olds: every fifth death in this age group was due to an accident on a two-wheeled vehicle.

**Table 3:** Proportion of two-wheeled vehicle related fatalities of all-cause mortality by age in 2008–19

Age, years	All deaths (*n*)	Two-wheeler fatalities (*n*)	Proportion of two-wheeler deaths (%)
10	55	2	3.6
11	55	1	1.8
12	58	4	6.9
13	77	4	5.2
14	93	6	6.5
15	145	30	20.7
16	185	31	16.8
17	221	23	10.4
18	377	8	2.1
19	384	8	2.1
20	441	8	1.8
21	459	9	2.0
22	473	4	0.8
23	456	3	0.7
24	500	6	1.2

## Discussion

During a 12-year study period, 147 adolescents died in bicycle, moped and motorcycle accidents in Finland. Most fatalities involved males and vehicles in suboptimal condition. Most accidents were collisions with another vehicle or other object. Head injuries were the most common cause of death and most adolescents died instantly at the scene of the accident. Among 15-year-olds, every fifth death was due an accident involving a two-wheeled vehicle.

Although mortality rates among adolescents have improved during the last decades, this positive change has been markedly slower than among younger children and especially slow among adolescent males.^2^ To reduce adolescent mortality, adolescent-specific issues require attention. Adolescents have a need for autonomous transportation but may lack the funds to invest in good-quality vehicles and protective gear. For example, none of the adolescents in this study had an airbag vest which is expensive but probably could have reduced some of the injuries.^23^

Besides financial issues, helmets and other protective gear may seem impractical to adolescents. In this study, nearly half of fatal accidents occurred in urban settings with speed limits of 50 km h^−1^ or less. These speeds may seem harmless, and probably routes in urban settings are familiar to riders of two-wheelers. The familiarity of surroundings may, however, increase mind wandering compared with unfamiliar situations^24^ and thus conversely increase accident risk. Although most accidents in our study occurred during daylight hours, lights and reflective or high-visibility clothing may still increase the visibility of two-wheelers to other road users.^25^

Bicycling holds great potential because it could be part of the solution for several of the vicious problems in modern society, namely environmental pollution, traffic congestion and the health problems related to a sedentary lifestyle. The European Commission has recently updated the Sustainable Urban Mobility Plans which aim to reduce pollution, congestion and accidents, and bicycling is a relevant part of these plans.^26^ In improving the safety of bicyclists, effective measures include safer speeds (30 km h^−1^), segregated bike lanes with intersections, mandatory bicycle lights and bicycle helmets.^25^

The last skills to mature in the adolescent brain are impulse control and response inhibition while the reward system is often hyper-responsive to rewards.^27^ These psychological rewards include thrill-seeking which makes adolescents more susceptible than adults to speeding and other risks in traffic.^28^ This was depicted in our results as peaks in fatalities at ages when adolescents acquire licenses to ride more powerful motorized two-wheelers. Adolescents do, however, already exhibit highly developed cognitive skills and the effect of increased speed on breaking distance and crash energy should be clearly demonstrated during driver’s education.^29^ The importance and credibility of speed limits^10^ should also be communicated to the parents of minors, since a fifth of all deaths among 15-year-olds were due to accidents on two-wheeled vehicles.

Mopeds and motorcycles have no legal requirement to be routinely inspected in Finland. Tuning of motorized two-wheelers is often a shared hobby of adolescent males and their fathers. Officially, any changes to the vehicles should be registered and checked for roadworthiness by officials. Adolescents and their parents often overlook this requirement because increasing motor power or maximum speed make mopeds illegal for their driver’s license class. Unfortunately, tuning of mopeds is especially dangerous because the brakes and chassis may be no match for the higher motor power and speed. Tires with inadequate pressure or worn flat further exacerbate the situation. Routine inspections may be the future due to EU directives which could potentially reduce the fatalities associated with two-wheelers.^30^

The greatest strength of our study is that it is based on national register data across a 12-year period. All fatal accidents have been examined following a published in-depth on-the-spot case-study method. The researchers also had access to the original case files and were thus able to double check, e.g. injury details and coroner’s reports. Our study is limited by the retrospective data collection, and we had no way of retrieving missing data. Regarding intentionality, we could not exclude suicidal behavior in this adolescent population. Transportation-related deaths and suicides are the leading causes of deaths among adolescents and sometimes conjoined. Despite the long study period, fatalities involving different types of two-wheelers were still fairly uncommon which limited the use of statistics. The fact that different multidisciplinary teams are responsible for investigating the accidents in different parts of Finland causes some subjectivity in the results, although the general methodology is the same. Despite its limitations, this study highlights several ways to improve road safety of adolescents.

In conclusion, among 147 fatal accidents involving adolescents and two-wheeled vehicles, vehicles had considerable technical flaws which may have affected the accident. Nearly half of victims lacked a properly attached helmet. Speeding and illegal tuning of motorized vehicles were common, and one-fifth of all-cause mortality among 15-year-olds was due to accidents involving two-wheeled vehicles. These findings can be directly utilized in the education of the public as well as new moped and motorcycle riders. Since males and females tended to exhibit different profiles of risky behavior, new targeted approaches may be useful to increase traffic safety of adolescents. One such approach could be connecting with the parents of adolescent males to enhance their understanding of the potential dangers of speeding and self-made chassis modifications.

## Supplementary Material

ckad198_Supplementary_Data

## Data Availability

The data underlying this article cannot be shared publicly due to the sensitive nature of the information. The data were provided by the Finnish Crash Data Institute by permission. Data may be shared by contacting the corresponding author and requesting special permission from the Finnish Crash Data Institute. The proper use of a helmet could save lives. The lack of proper maintenance of the two-wheeled motor vehicles contributes to major percentage of the fatal accidents. Risk-taking-behavior—leading to for example speeding—could be better addressed during drivers’ education and communicated to parents.

## References

[1] Sawyer SM , Azzopardi PS, Wickremarathne D, Patton GC. The age of adolescence. Lancet Child Adolesc Health 2018;2:223–8. 30169257 10.1016/S2352-4642(18)30022-1

[2] Ward JL , Azzopardi PS, Francis KL, et al Global, regional, and national mortality among young people aged 10-24 years, 1950-2019: a systematic analysis for the Global Burden of Disease Study 2019. Lancet 2021;398:1593–618. 34755628 10.1016/S0140-6736(21)01546-4 PMC8576274

[3] Centers for Disease Control and Prevention. Teen Drivers: Get the Facts. October 2021. Available at: https://www.cdc.gov/transportationsafety/teen_drivers/teendrivers_factsheet.html (11 November 2022, date last accessed).

[4] Eurostat: Statistics Explained. Being Young in Europe Today—Health. Available at: https://ec.europa.eu/eurostat/statistics-explained/index.php?title=Being_young_in_Europe_today_-_health#Causes_of_death (11 November 2022, date last accessed).

[5] Viner R , Booy R. Epidemiology of health and illness. BMJ 2005;330:411–4. 15718543 10.1136/bmj.330.7488.411 PMC549118

[6] European Transport Safety Council. Reducing Road Deaths among Young People Aged 15 to 30. PIN Flash Report 41. October 2021. Available at: https://etsc.eu/wp-content/uploads/PIN-Flash-41_web_FINAL.pdf (11 November 2022, date last accessed).

[7] European Road Safety Observatory. SafetyNet. Available at: http://www.dacota-project.eu/Links/erso/safetynet/content/safetynet.html (11 November 2022, date last accessed).

[8] Lardelli-Claret P , Jiménez-Moleón JJ, de Dios Luna-del-Castillo J, et al Driver dependent factors and the risk of causing a collision for two wheeled motor vehicles. Inj Prev 2005;11:225–31. 16081752 10.1136/ip.2004.006957 PMC1730254

[9] Moller M , Haustein S. Factors contributing to young moped rider accidents in Denmark. Accid Anal Prev 2016;87:1–7. 26619285 10.1016/j.aap.2015.11.008

[10] European Transport Safety Council. How Safe Is Walking and Cycling in Europe? PIN Flash Report 38, 2020. Available at: https://etsc.eu/category/publications/ (31 July 2023, date last accessed).

[11] Lenton S , Finlay FO. Public health approaches to safer cycling for children based on developmental and physiological readiness: implications for practice. BMJ Paediatr Open 2018;2:e000123. 10.1136/bmjpo-2017-000123 PMC5887771 29637180

[12] Unkuri J , Salminen P, Kallio P, Kosola S. Teens on wheels and consequences: a six-year population-based study of bicycle and moped injuries. Eur J Pediatr Surg 2021;31:266–72. 32526781 10.1055/s-0040-1712930

[13] The Motorcycle Industry in Europe. Market Data. Available at: https://www.acem.eu/market-data (11 November 2022, date last accessed).

[14] Kosola S , Salminen P, Kallio P. Driver's education may reduce annual incidence and severity of moped and scooter accidents. A population-based study. Injury 2016;47:239–43. 26606989 10.1016/j.injury.2015.10.074

[15] Federation of European Motorcyclists’ Associations. Riderscan Report. Available at: https://www.cieca.eu/sites/default/files/public-pages/Projects/riderscan_report.pdf (11 November 2022, date last accessed).

[16] Driving Licence Act 386/2011 Finland. Available at: https://www.finlex.fi/fi/laki/ajantasa/2011/20110386 Available in Finnish. (11 November 2022, date last accessed).

[17] Salo I , Parkkari K, Sulander P, Keskinen E. In-depth on-the-spot road accident investigation in Finland. In: 2nd International Conference on ESAR “Expert Symposium on Accident Research” 2007. Available at: https://edocs.tib.eu/files/e01fn19/1678914894.pdf (11 November 2022, date last accessed).

[18] Act on the Investigation of Road and Cross-country Traffic Accidents 1512/2016 Finland. Available at: https://www.finlex.fi/fi/laki/alkup/2016/20161512 (available in Finnish) (11 November 2022, date last accessed).

[19] Paul D . VALT Method 2003. Finnish Motor Insurerś Centre, Road Accident Investigation Delegation 2004. Helsinki, Finland.

[20] Baker SP , O’Neill B, Haddon W Jr., Long WB. The injury severity score: a method for describing patients with multiple injuries and evaluating emergency care. J Trauma 1974;14:187–96. 4814394

[21] Colnaric JM , El Sibai RH, Bachir RH, El Sayed MJ. Injury severity score as a predictor of mortality in adult trauma patients by injury mechanism types in the United States: a retrospective observational study. Medicine (Baltimore) 2022;101:e29614. 35839012 10.1097/MD.0000000000029614 PMC11132402

[22] Brown JB , Gestring ML, Leeper CM, et al The value of the Injury Severity Score in pediatric trauma: time for a new definition of severe injury? J Trauma Acute Care Surg 2017;82:995–1001. 28328674 10.1097/TA.0000000000001440 PMC5464600

[23] Giustini M , Cedri S, Tallon M, et al Use of back protector device on motorcycles and mopeds in Italy. Int J Epidemiol 2014;43:1921–8. 25342252 10.1093/ije/dyu209

[24] Harms IM , Burdett BRD, Charlton SG. The role of route familiarity in traffic participants’ behaviour and transport psychology research: a systematic review. Transport Res Interdisc Persp 2021;9:100331.

[25] World Health Organization: Cyclist Safety: An Information Resource for Decision-makers and Practitioners. Geneva, 2020. Available at: https://www.who.int/publications/i/item/cyclist-safety-an-information-resource-for-decision-makers-and-practitioners (31 July 2023, date last accessed).

[26] Rupprecht Consult, editor. *Guidelines for Developing and Implementing a Sustainable Urban Mobility Plan*, 2nd edn. 2019. Available at: https://www.eltis.org/mobility-plans/sump-guidelines (31 July 2023, date last accessed).

[27] Blakemore SJ , Robbins TW. Decision-making in the adolescent brain. Nat Neurosci 2012;15:1184–91. 22929913 10.1038/nn.3177

[28] Galvan A . The teenage brain: sensitivity to rewards. Curr Dir Psychol Sci 2013;22:88–93. 10.1177/0963721413476512 PMC3992953 24761055

[29] Insurance Institute for Highway Safety. Speed. 2022. Available at: https://www.iihs.org/topics/speed (11 November 2022, date last accessed).

[30] Directive 2014/45/EU of the European Parliament and of the Council of 3 April 2014 on Periodic Roadworthiness Tests for Motor Vehicles and Their Trailers and Repealing Directive 2009/40/EC. Available at: https://eur-lex.europa.eu/legal-content/EN/TXT/?uri=celex%3A32014L0045 (11 November 2022, date last accessed).

