# Supplementary material for: On a collision course: fatal motorcycle and bicycle accidents of adolescents in Finland from 2008 to 2019
Source: Eur J Public Health. 2023 Nov 8;34(2):267–71. doi: 10.1093/eurpub/ckad198 (PMC10990557; doi:10.1093/eurpub/ckad198)
Supplement: ckad198_Supplementary_Data [file ckad198_supplementary_data.zip › ckad198_Supplementary_Data/ejph-2023-01-om-0042-File002.docx]

**Table supplementary. Accident types, injuries, and treatment details.**

|  | Bicycle (n=20) | Moped (n=50) | Motorcycle <125cc (n=44) | Motorcycle >125cc (n=33) | Total (n=147) |
| --- | --- | --- | --- | --- | --- |
| Accident type, n (%) |  |  |  |  |  |
| Collision with another vehicle | 18 (90) | 41 (82) | 24 (55) | 16 (49) | 99 (67) |
| Collision with other object | 0 | 7 (14) | 17 (39) | 11 (33) | 35 (24) |
| Driving off road or fall without collision | 2 | 2 (4) | 3 (7) | 6 (18) | 13 (9) |
| Site of most severe injury, n (%) |  |  |  |  |  |
| Head | 12 (60) | 31 (65) | 29 (67) | 17 (52) | 89 (62) |
| Thorax | 3 (15) | 12 (25) | 12 (28) | 12 (36) | 39 (27) |
| Spine | 4 (20) | 2 (4) | 2 (5) | 2 (6) | 10 (7) |
| Abdomen | 0 | 3 (6) | 0 | 2 (6) | 5 (4) |
| ISS, n (%) |  |  |  |  |  |
| 0-15 | 1 (5) | 2 (4) | 0 | 0 | 3 (2) |
| 16-24 | 1 (5) | 1 (2) | 2 (5) | 0 | 4 (3) |
| 25-74 | 9 (45) | 27 (54) | 18 (41) | 12 (36) | 66 (45) |
| 75 (max) | 9 (45) | 19 (38) | 23 (52) | 21 (64) | 72 (49) |
| Operative treatment, n (%) | 3 (15) | 10 (20) | 4 (9) | 2 (6) | 19 (13) |
| Time from accident to death, n (%) |  |  |  |  |  |
| Instant | 7 (35) | 26 (52) | 27 (61) | 22 (67) | 82 (56) |
| Before first aid/treatment | 4 (20) | 7 (14) | 5 (11) | 5 (15) | 21 (14) |
| In 6 hours | 2 (10) | 8 (16) | 4 (9) | 4 (12) | 18 (12) |
| In 24 hours | 4 (20) | 2 (4) | 3 (7) | 1 (3) | 10 (7) |
| In 7 days | 3 (15) | 7 (14) | 5 (11) | 1 (3) | 16 (11) |
| Place of death, n (%) |  |  |  |  |  |
| At scene | 11 (55) | 33 (66) | 32 (73) | 27 (82) | 103 (70) |
| During transportation | 0 | 1 (2) | 1 (2) | 1 (3) | 3 (2) |
| In hospital | 9 (45) | 16 (32) | 11 (25) | 5 (15) | 41 (28) |

ISS = Injury Severity Score.

Data on ISS missing in two cases (one bicycle and one motorcycle <125cc).
