# Supplementary figures and images for: On a collision course: fatal motorcycle and bicycle accidents of adolescents in Finland from 2008 to 2019
Source: Eur J Public Health. 2023 Nov 8;34(2):267–71. doi: 10.1093/eurpub/ckad198 (PMC10990557; doi:10.1093/eurpub/ckad198)

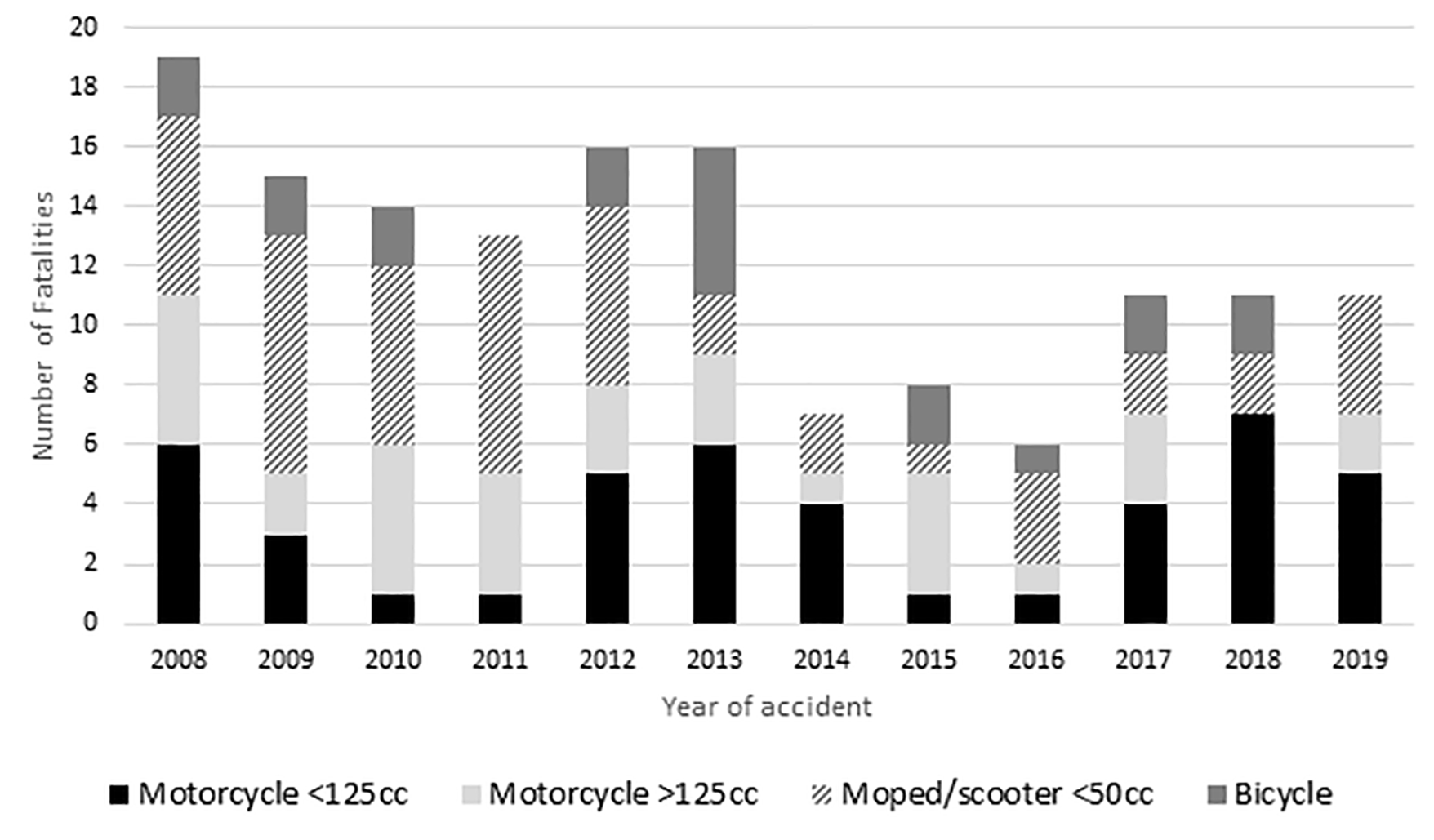

Supplement: ckad198_Supplementary_Data [file ckad198_supplementary_data.zip › ckad198_Supplementary_Data/ejph-2023-01-om-0042-File004.tif]
